# Long-term Efficacy of Neoadjuvant Chemoradiotherapy Plus Surgery for the Treatment of Locally Advanced Esophageal Squamous Cell Carcinoma: The NEOCRTEC_50_10 Randomized Clinical Trial

**DOI:** 10.1001/jamasurg.2021.2373

**Published:** 2021-06-23

**Authors:** Hong Yang, Hui Liu, Yuping Chen, Chengchu Zhu, Wentao Fang, Zhentao Yu, Weimin Mao, Jiaqing Xiang, Yongtao Han, Zhijian Chen, Haihua Yang, Jiaming Wang, Qingsong Pang, Xiao Zheng, Huanjun Yang, Tao Li, Xu Zhang, Qun Li, Geng Wang, Baofu Chen, Teng Mao, Min Kong, Xufeng Guo, Ting Lin, Mengzhong Liu, Jianhua Fu

**Affiliations:** 1 Department of Thoracic Surgery, Sun Yat-sen University Cancer Center, State Key Laboratory of Oncology in South China, Guangdong Esophageal Cancer Institute, Collaborative Innovation Center for Cancer Medicine, Guangzhou, China; 2 Department of Radiation Oncology, Sun Yat-sen University Cancer Center, State Key Laboratory of Oncology in South China, Guangdong Esophageal Cancer Institute, Collaborative Innovation Center for Cancer Medicine, Guangzhou, China; 3 Department of Thoracic Surgery, Cancer Hospital of Shantou University Medical College, Shantou, China; 4 Department of Thoracic Surgery, Taizhou Hospital, Wenzhou Medical University, Linhai, China; 5 Department of Thoracic Surgery, Shanghai Chest Hospital, Shanghai Jiaotong University, Shanghai, China; 6 Department of Thoracic Surgery, National Cancer Center, National Clinical Research Center for Cancer, Cancer Hospital and Shenzhen Hospital, Chinese Academy of Medical Sciences and Peking Union Medical College, Shenzhen, China; 7 Tianjin Medical University Cancer Hospital, Tianjin, China; 8 Department of Thoracic Surgery, Zhejiang Cancer Hospital, Hangzhou, China; 9 Department of Thoracic Surgery, Fudan University Shanghai Cancer Center, Shanghai, China; 10 Department of Thoracic Surgery, Sichuan Cancer Hospital, Chengdu, China; 11 Department of Radiation Oncology, Cancer Hospital of Shantou University Medical College, Shantou, China; 12 The University of Hong Kong–Shenzhen Hospital, Shenzhen, China; 13 Department of Radiation Oncology, Taizhou Hospital, Wenzhou Medical University, Linhai, China; 14 Department of Radiation Oncology, Shanghai Chest Hospital, Shanghai Jiaotong University, Shanghai, China; 15 Department of Radiation Oncology, Tianjin Medical University Cancer Hospital, Tianjin, China; 16 Department of Radiation Oncology, Zhejiang Cancer Hospital, Hangzhou, China; 17 Department of Radiation Oncology, Fudan University Shanghai Cancer Center, Shanghai, China; 18 Department of Radiation Oncology, Sichuan Cancer Hospital, Chengdu, China

## Abstract

**Importance:**

The prognosis of patients with locally advanced esophageal squamous cell carcinoma (ESCC) remains poor after surgery. Neoadjuvant chemoradiotherapy (NCRT) has been shown to potentially improve survival.

**Objective:**

To compare the treatment efficacy of NCRT plus surgery with surgery alone for long-term survival among patients with locally advanced ESCC.

**Design, Setting, and Participants:**

The Neoadjuvant Chemoradiotherapy for Esophageal Cancer 5010 study was a multicenter open-label randomized phase 3 clinical trial that enrolled patients between June 1, 2007, and December 31, 2014. Follow-up ended on December 31, 2019. The study was conducted at 8 centers in China. A total of 451 patients aged 18 to 70 years with thoracic ESCC stage T1-4N1M0/T4N0M0 were enrolled and randomized. Data were analyzed from December 1, 2019, to June 30, 2020.

**Interventions:**

Patients randomized to receive NCRT plus surgery (NCRT group) received preoperative chemotherapy (25 mg/m^2^ of vinorelbine on days 1 and 8 and 75 mg/m^2^ of cisplatin on day 1 or 25 mg/m^2^ of cisplatin on days 1 to 4) every 3 weeks for 2 cycles and concurrent radiotherapy (40.0 Gy, administered in 20 fractions of 2.0 Gy for 5 days per week) followed by surgery. Patients randomized to receive surgery alone (surgery group) underwent surgery after randomization.

**Main Outcomes and Measures:**

The primary end point was overall survival in the intention-to-treat population. The secondary end point was disease-free survival.

**Results:**

A total of 451 patients (mean [SD] age, 56.5 [7.0] years; 367 men [81.4%]) were randomized to the NCRT (n = 224) and surgery (n = 227) groups and were eligible for the intention-to-treat analysis. By December 31, 2019, 224 deaths had occurred. The median follow-up was 53.5 months (interquartile range, 18.2-87.4 months). Patients receiving NCRT plus surgery had prolonged overall survival compared with those receiving surgery alone (hazard ratio, 0.74; 95% CI, 0.57-0.97; *P* = .03), with a 5-year survival rate of 59.9% (95% CI, 52.9%-66.1%) vs 49.1% (95% CI, 42.3%-55.6%), respectively. Patients in the NCRT group compared with the surgery group also had prolonged disease-free survival (hazard ratio, 0.60; 95% CI, 0.45-0.80; *P* < .001), with a 5-year survival rate of 63.6% (95% CI, 56.0%-70.2%) vs 43.0% (95% CI, 36.0%-49.7%), respectively.

**Conclusions and Relevance:**

In this randomized clinical trial, treatment with NCRT plus surgery significantly improved long-term overall survival and disease-free survival and therefore may be considered a standard of care for patients with locally advanced ESCC.

**Trial Registration:**

ClinicalTrials.gov Identifier: NCT01216527

## Key Points

**Question:**

Does treatment with neoadjuvant chemoradiotherapy plus surgery improve the long-term survival of patients with locally advanced esophageal squamous cell carcinoma (ESCC) compared with surgery alone?

**Findings:**

In this randomized clinical trial of 451 patients with locally advanced ESCC, treatment with neoadjuvant chemoradiotherapy plus surgery showed significantly improved 5-year overall survival of 59.9% compared with 49.1% for surgery alone, as well as improved disease-free survival.

**Meaning:**

In this study, long-term outcomes demonstrated a survival benefit from neoadjuvant chemoradiotherapy followed by surgery compared with surgery alone for the treatment of patients with locally advanced ESCC, indicating that this combination may be considered a standard of care in this patient population.

## Teaser

This randomized clinical trial provides updated outcomes of the Neoadjuvant Chemoradiotherapy for Esophageal Cancer 5010, which examined the long-term efficacy of neoadjuvant chemoradiotherapy plus surgery vs surgery alone for the treatment of patients with esophageal squamous cell carcinoma.

## Introduction

Esophageal cancer is the ninth most frequent cancer and the sixth leading cause of cancer-associated death in the world.^1^ More than 50% of patients with esophageal cancer reside in East Asia, and 90% of those patients have esophageal squamous cell carcinoma (ESCC).^2^ For patients with locally advanced esophageal cancer, surgery remains the mainstay of current therapy. However, patients with locally advanced esophageal cancer who undergo surgery with no additional therapies have a low 5-year survival rate of only 25%.^3,4^ Although recent evidence has suggested that neoadjuvant chemoradiotherapy (NCRT) plus surgery could prolong the overall survival (OS) of patients with locally advanced esophageal cancer,^5^ its role in the treatment of patients with ESCC remains controversial.^4,6,7,8,9,10,11,12,13^ Therefore, identification of the efficacy of this multidisciplinary treatment for locally advanced ESCC is an important current research topic.

The Neoadjuvant Chemoradiotherapy for Esophageal Cancer 5010 (NEOCRTEC_50_10) randomized clinical trial^14^ compared treatment with NCRT followed by surgery (NCRT group) vs treatment with surgery alone (surgery group). The clinical trial included 451 patients with locally advanced ESCC from 8 Chinese centers between June 1, 2007, and December 31, 2014. Initial results were reported in 2018 after a minimum follow-up of 24 months (NCRT group: median follow-up, 41.0 months; interquartile range [IQR], 20.1-59.3 months; surgery group: median follow-up, 34.6 months; IQR, 17.7-54.2 months). The results showed significant benefits in both median OS and disease-free survival (DFS) in favor of the NCRT group. The NCRT group experienced a significantly increased margin-negative (R0) resection rate (98.4%) compared with the surgery group (91.2%; *P* = .002). In the NCRT group, 185 of 224 patients (82.6%) completed the multimodal therapy, with a pathologic complete response rate of 43.2%. With respect to incidences of postoperative complications, no significant differences were observed between the groups, with the exception of arrhythmia (13% for the NCRT group vs 4.0% for the surgery group; *P* = .001). The anastomotic leak rates were 8.6% for the NCRT group and 12.3% for the surgery group (*P* = .23). The peritreatment mortality rates of the 2 groups were similar (2.2% in the NCRT group vs 0.4% in the surgery group; *P* = .21).

The current study aimed to compare the treatment efficacy of NCRT plus surgery with surgery alone for long-term survival among patients with locally advanced ESCC. Updated outcomes are reported to provide insight into the durability of the efficacy of NCRT plus surgery after a longer follow-up period.

## Methods

### Patients

As previously described elsewhere,^14^ patients with stage T1-4N1M0/T4N0M0 (based on American Joint Committee on Cancer, sixth edition, staging criteria^15^) histologically proven and potentially resectable thoracic ESCC were enrolled. Patients eligible for enrollment were aged 18 to 70 years; had a Karnofsky performance score of 90 or higher; and had adequate bone marrow, hepatic, and kidney function. Patients were excluded if they had a history of other cancers (including skin cancers), a history of gastrectomy leading to infeasible utility of gastric conduit for reconstruction, or severe comorbidities contraindicating surgery. The study was approved by the ethics committees or institutional review boards of the 8 participating centers in China. Each patient provided written informed consent before randomization. This study followed the Consolidated Standards of Reporting Trials (CONSORT) reporting guideline for randomized clinical trials.

### Study Design and End Points

The NEOCRTEC_50_10 study was a multicenter open-label phase 3 randomized clinical trial. The primary end point was OS, which was defined as the time from the date of randomization to the date of death or last follow-up. The secondary end point was DFS, which was defined as the time from the date of R0 resection to the date of recurrence or death. Disease recurrence included locoregional recurrence (LRR) and distant recurrence (DR) that occurred after R0 resection. Locoregional recurrence was defined as relapse within the esophagus or regional lymph nodes, excluding the supraclavicular nodes. Distant recurrence was defined as diffused disease in distant sites beyond the locoregional sites. Adverse events associated with chemoradiotherapy were graded according to the National Cancer Institute’s *Common Terminology Criteria for Adverse Events*, version 3.^16^

### Randomization

Patients were randomized in a 1:1 ratio to receive NCRT plus surgery or surgery alone using computer-generated random numbers provided by the Sun Yat-sen University Cancer Center Clinical Trial Center. A stratified permuted-block method was adopted, with the stratification based on participating centers and a permuted block size of 20.

### Procedures

Clinical staging was determined by the following modalities: computed tomography of the neck, chest, and upper abdomen with intravenous contrast; esophagogastroduodenoscopy; endoscopic ultrasonography; and ultrasonography of the neck. Suspicious tumorous involvement of the trachea or bronchi was further assessed through bronchoscopy. Positron emission tomography and radionuclide bone scans were optional.

The current clinical trial protocol (Supplement 1) followed the same preoperative chemoradiotherapy protocol used in a previous phase 2 clinical trial.^17^ In that study, R0 resection was successfully completed in 98% of patients, with possibly improved prognosis. Patients in the NCRT group received either 25 mg/m^2^ of vinorelbine on days 1 and 8 and 75 mg/m^2^ of cisplatin on day 1 or 25 mg/m^2^ of cisplatin on days 1 to 4 of a 21-day cycle for 2 cycles. Concurrent radiotherapy started on the first day of chemotherapy. The gross tumor volume encompassed the primary tumor and enlarged regional lymph nodes. The clinical target volume included the gross tumor volume with an additional 3.0-cm proximal and distal margin and 0.5- to 1.0-cm radial margin to cover the area of subclinical involvement. The planning target volume was expanded to include an 0.8-cm margin from the clinical target volume for setup variations and respiratory-induced tumor motion. Radiotherapy was delivered with 6- to 8-megavoltage photons using the 3-dimensional conformal radiotherapy technique. The total planned dose for the planning target volume was 40.0 Gy in 20 fractions over 4 weeks.

Tumors were restaged through endoscopic ultrasonography and computed tomography of the neck, chest, and upper abdomen. McKeown or Ivor Lewis esophagectomy with 2-field lymphadenectomy (eMethods in Supplement 2) was performed 4 to 6 weeks after the completion of NCRT. Bilateral recurrent nerve lymph node dissection was mandatory. Both open and minimally invasive esophagectomy were used. Patients in the surgery group received surgery after randomization. Clinic follow-up visits occurred once every 3 months within the first year and once every 6 months thereafter.

### Statistical Analysis

Based on a previous phase 2 study,^17^ the current study was estimated to detect an improvement in the median OS of 39 months for the surgery group to 56 months for the NCRT group (HR, 0.72) with a 2-sided significance level of α = .05 and 80% statistical power. An estimated sample of 430 participants (215 per group) was required with a 7-year accrual and 2 years of follow-up assuming an attrition rate of 10%. Two interim analyses were performed by an independent safety and data monitoring committee on June 1, 2011, and December 31, 2015, after 123 patients and 451 patients had been enrolled, respectively. The significance threshold was α = .000527 for the first interim analysis, α = .014 for the second analysis, and α = .045 for the final analysis, defined by the O’Brien-Fleming boundary.

The analysis database was locked on December 31, 2019. Survival was estimated using the Kaplan-Meier method, and a log-rank test was used for comparisons between groups. The analysis of OS was based on the intention-to-treat population. A secondary population comprising a cohort of patients who had undergone R0 resection was included in the DFS analysis. We applied univariable and multivariable Cox regression models to estimate the effect of NCRT among subgroups according to age, sex, tumor location, clinical tumor category (T0-T4), and clinical nodal category (N0-N3). Hazard ratios and 95% CIs were calculated.

To investigate the recurrence pattern for the secondary population, we calculated the proportion of patients with disease recurrence (including LRR, DR, and both) at different points after R0 resection. Patients who were identified as having LRR or DR were censored for the other type of recurrence, and patients who were diagnosed with both LRR and DR simultaneously were documented as having both types. Deaths without recurrence were also censored. Locoregional, distant, and overall recurrences were analyzed separately using a univariate Cox model, and HRs were calculated. The proportional hazards assumption was confirmed based on Schoenfeld residuals.^18^ A number-needed-to-treat analysis was also conducted.

All statistical analyses were performed using SAS software, version 9.4 (SAS Institute). Data were analyzed from December 1, 2019, to June 30, 2020.

## Results

### Patients and Treatment

**Figure 1.**
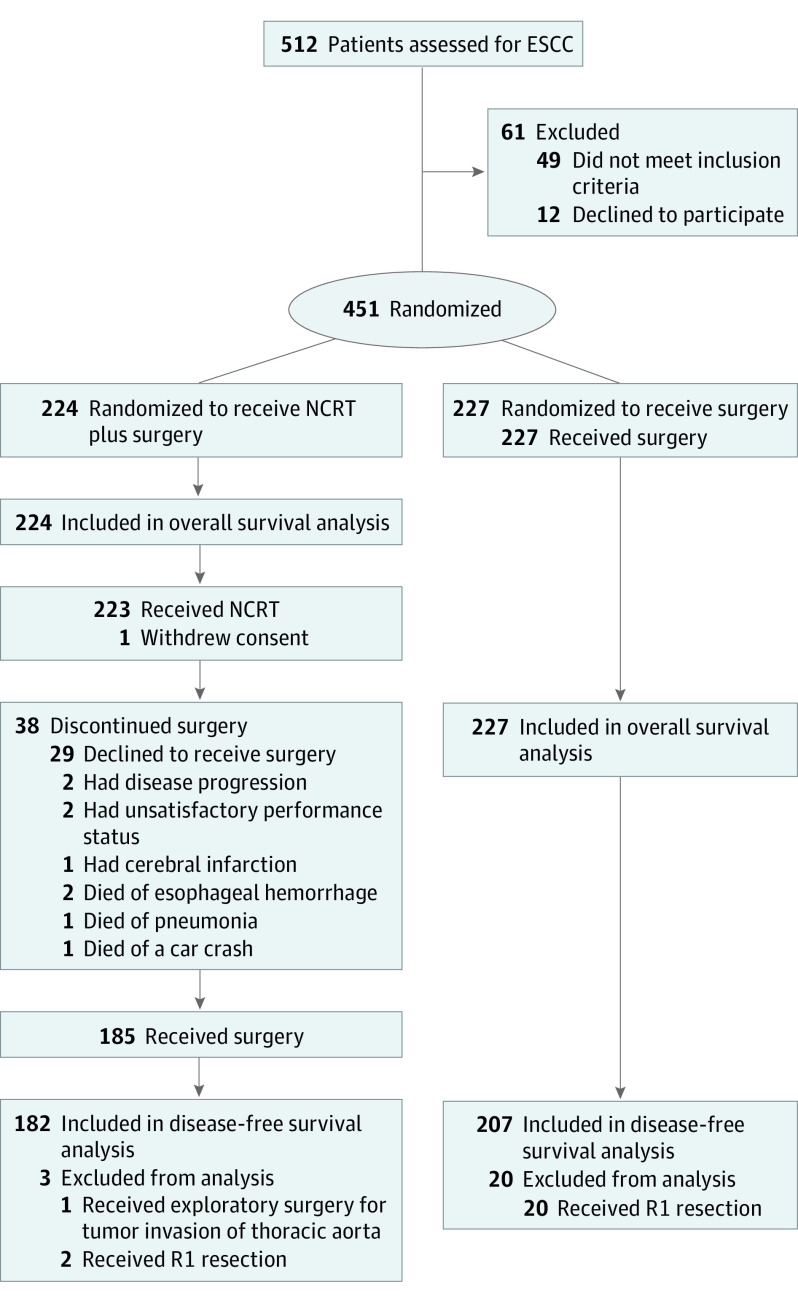
CONSORT Flow Diagram ESCC indicates esophageal squamous cell carcinoma; NCRT, neoadjuvant chemoradiotherapy.

Between June 2007 and December 2014, a total of 451 patients with ESCC (mean [SD] age, 56.5 [7.0] years; 367 men [81.4%]) from 8 institutions in China (eTable 1 in Supplement 2) were randomized to receive NCRT plus surgery (n = 224) or surgery alone (n = 227) (Figure 1). The demographic and clinicopathologic characteristics were well balanced between the NCRT group (mean [SD] age, 56.0 [7.1] years; 190 men [84.8%]; 188 individuals [83.9%] with stage III disease) and the surgery group (mean [SD] age, 56.9 [6.9] years; 177 men [78.0%]; 190 individuals [83.7%] with stage III disease) (Table 1). Among the total population, a majority of patients had T3 tumors (272 patients [60.3%]) and most patients had N1 nodes (390 patients [86.5%]).

**Table 1.** Baseline Patient Characteristics

Characteristic	No. (%)	
	NCRT group (n = 224)	Surgery group (n = 227)
Age, y		
Mean (SD) [range]	56.0 (7.1) [31-70]	56.9 (6.9) [35-70]
≤60	165 (73.7)	154 (67.8)
>60	59 (26.3)	73 (32.2)
Sex		
Male	190 (84.8)	177 (78.0)
Female	34 (15.2)	50 (22.0)
BMI, mean (SD)	22.2 (2.9)	22.0 (3.5)
<18.5	21 (9.4)	27 (11.9)
18.5-24.9	170 (75.9)	166 (73.1)
25.0-29.9	29 (12.9)	31 (13.7)
≥30	4 (1.8)	3 (1.3)
Tumor location		
Proximal third	26 (11.6)	22 (9.7)
Middle third	158 (70.5)	160 (70.5)
Distal third	40 (17.9)	45 (19.8)
Surgery mode		
Resection		
R0	182 (81.3)	207 (91.2)
R1	2 (0.9)	20 (8.8)
Exploratory	1 (0.4)	0
No surgery	39 (17.4)	0
Clinical T category		
1	1 (0.4)	1 (0.4)
2	35 (15.6)	35 (15.4)
3	123 (54.9)	149 (65.6)
4	65 (29.0)	42 (18.5)
Clinical N category		
0	34 (15.2)	27 (11.9)
1	190 (84.8)	200 (88.1)
Clinical stage group		
IIB	36 (16.1)	37 (16.3)
III	188 (83.9)	190 (83.7)

Abbreviations: BMI, body mass index (calculated as weight in kilograms divided by height in meters squared); NCRT, neoadjuvant chemoradiotherapy; N category, nodal category; R0, margin-negative resection; R1, margin-positive resection for microscopic residual tumor; T category, tumor category.

Treatment adherence and safety profiles have been shown in a previous article.^14^ In brief, among 224 patients in the NCRT group, 195 patients (87.1%) received 2 cycles of chemotherapy, and 28 patients (12.5%) received only 1 cycle. Almost all patients (222 individuals [99.1%]) received total-dose radiotherapy as specified in the protocol, with the exception of 1 patient who received only 22.0 Gy because of death associated with pneumonia. Grade 3 or 4 adverse events comprised both hematologic toxic effects, which were observed in 121 of 223 patients (54.3%), and nonhematologic toxic effects, which were observed in 16 of 223 patients (7.2%); among those with nonhematologic toxic effects, leukopenia and neutropenia had the highest prevalence, occurring in 109 patients (48.9%) and 102 patients (45.7%), respectively. A total of 185 of 224 patients (82.6%) received surgery after NCRT; of those, 182 patients (98.4%) underwent R0 resection.

With regard to the surgery group, R0 resection was performed in 207 of 227 patients (91.2%). A total of 20 patients (8.8%) underwent margin-positive (R1) resection and received postoperative chemoradiotherapy.

### Events During Follow-up

By December 31, 2019, the median follow-up time of all surviving patients was 53.5 months (range, 0.9-149.2 months; IQR, 18.2-87.4 months). Median follow-up time was 63.7 months (IQR, 20.0-91.4 months) in the NCRT group and 39.3 months (17.4-83.3 months) in the surgery group. A total of 224 deaths (49.7%) were recorded during follow-up; of those, 177 deaths (79.0%) were associated with cancer. Five deaths (2.2%) were treatment-associated; 4 of those deaths occurred in the NCRT group (1 patient died during receipt of NCRT, 2 died during the interval between chemoradiotherapy and surgery, and 1 died postoperatively), and 1 occurred in the surgery group. Forty-two patients (18.8%) died of conditions not associated with cancer or treatment, including 22 deaths in the NCRT group and 20 deaths in the surgery group. Among 38 of 224 patients (17.0%) who did not receive surgery after chemoradiotherapy, 4 patients died while waiting for surgery. Overall, the postoperative mortality rate in the study population was 0.9%.

### Survival

**Figure 2.**
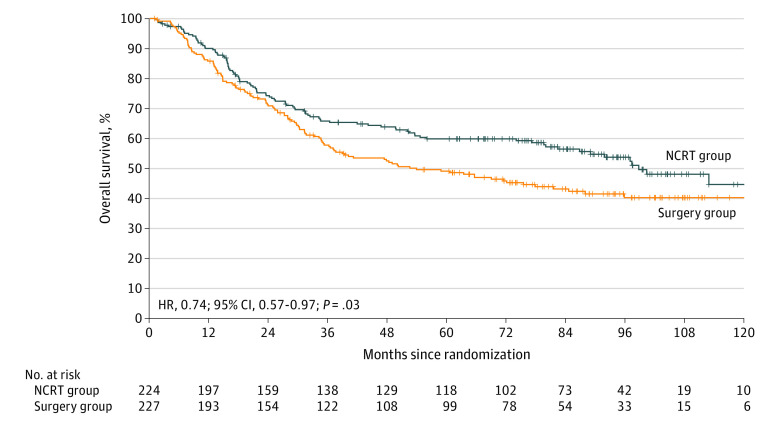
Overall Survival Overall survival for the intention-to-treat population. HR indicates hazard ratio; NCRT, neoadjuvant chemoradiotherapy.

In total, 100 of 224 deaths (44.6%) occurred in the NCRT group, and 124 of 227 deaths (54.6%) occurred in the surgery group. Overall survival was significantly improved among patients in the NCRT group compared with those in the surgery group (HR, 0.74; 95% CI, 0.57-0.97; *P* = .03) (Figure 2). Overall survival rates at 3 and 5 years were 65.8% (95% CI, 59.1%-71.7%) and 59.9% (95% CI, 52.9%-66.1%) in the NCRT group compared with 57.8% (95% CI, 51.0%-64.0%) and 49.1% (95% CI, 42.3%-55.6%) in the surgery group, with HRs of 0.77 (95% CI, 0.57-1.04; *P* = .09) at 3 years and 0.73 (95% CI, 0.55-0.97; *P* = .03) at 5 years. The absolute OS benefit at 5 years was 10.8%, and the number needed to treat was 9.3 (95% CI, 5.0-73.2).

**Figure 3.**
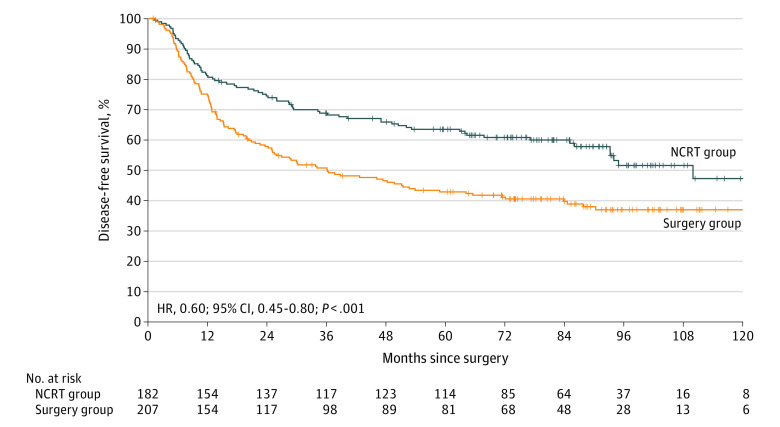
Disease-Free Survival Disease-free survival for patients with margin-zero resection. HR indicates hazard ratio; NCRT, neoadjuvant chemoradiotherapy.

Of 389 R0 resections, 77 of 182 relapses (42.3%) occurred in the NCRT group, and 123 of 207 relapses (59.4%) occurred in the surgery group. The NCRT group also experienced significantly improved DFS compared with the surgery group (HR, 0.60; 95% CI, 0.45-0.80; *P* < .001) (Figure 3). The DFS rates at 3 and 5 years were 68.9% (95% CI, 61.5%-75.1%) and 63.6% (95% CI, 56.0%-70.2%) in the NCRT group compared with 50.3% (95% CI, 43.2%-57.0%) and 43.0% (95% CI, 36.0%-49.7%) in the surgery group, with HRs of 0.55 (95% CI, 0.40-0.77; *P* < .001) at 3 years and 0.55 (95% CI, 0.40-0.74; *P* < .001) at 5 years. The absolute DFS benefit at 5 years was 20.6%, and the number needed to treat was 4.9 (95% CI, 3.3-9.4). Subgroup analyses for OS and DFS verified the favorable treatment effect of NCRT (Table 2; eTable 2 in Supplement 2).

**Table 2.** Univariable and Multivariable Cox Analyses for Overall Survival Stratified by Subgroup

Variable	Events, No./total No. (%)		Univariable analysis		Multivariable analysis	
	NCRT group	Surgery group	HR (95% CI)	*P* value for interaction	HR (95% CI)	*P* value for interaction
All patients	100/224 (44.6)	124/227 (54.6)	0.74 (0.57-0.97)	NA	0.69 (0.53-0.91)	NA
Age, y						
≤60	70/165 (42.4)	84/154 (54.5)	0.71 (0.52-0.98)	.51	0.65 (0.47-0.90)	.52
>60	30/59 (50.8)	40/73 (54.8)	0.83 (0.52-1.35)		0.75 (0.46-1.24)	
Sex						
Male	89/190 (46.8)	98/177 (55.4)	0.79 (0.59-1.05)	.21	0.75 (0.56-1.00)	.27
Female	11/34 (32.4)	26/50 (52.0)	0.46 (0.22-0.96)		0.46 (0.21-0.97)	
Tumor location						
Proximal third	13/26 (50.0)	14/22 (63.6)	0.56 (0.26-1.23)	.75	0.49 (0.21-1.14)	.62
Middle third	67/158 (42.4)	80/160 (50.0)	0.79 (0.57-1.09)		0.76 (0.55-1.06)	
Distal third	20/40 (50.0)	30/45 (66.7)	0.64 (0.36-1.15)		0.55 (0.29-1.03)	
Clinical T category						
1-2	15/36 (41.7)	14/36 (38.9)	1.22 (0.59-2.54)	.18	1.22 (0.58-2.57)	.17
3	50/123 (40.7)	85/149 (57.0)	0.59 (0.41-0.84)		0.57 (0.40-0.81)	
4	35/65 (53.8)	25/42 (59.5)	0.80 (0.48-1.34)		0.80 (0.47-1.35)	
Clinical N category						
0	17/34 (50.0)	15/27 (55.6)	0.66 (0.33-1.33)	.88	0.67 (0.31-1.47)	.73
1	83/190 (43.7)	109/200 (54.5)	0.74 (0.56-0.98)		0.70 (0.52-0.93)	

Abbreviations: HR, hazard ratio; NA, not applicable; NCRT, neoadjuvant chemoradiotherapy; N category, nodal category; T category, tumor category.

### Recurrence

Compared with surgery alone, NCRT plus surgery reduced both LRR and DR in patients with locally advanced ESCC (eTable 3 in Supplement 2). Of 182 patients with R0 resection in the NCRT group, 25 patients (13.7%) experienced LRR, 46 patients (25.3%) experienced DR, and 8 patients (4.4%) experienced concurrent LRR and DR at the first time of recurrence confirmation. Of 207 patients with R0 resection in the surgery group, 45 patients (21.7%) experienced LRR, 74 patients (35.7%) experienced DR, and 17 patients (8.2%) experienced concurrent LRR and DR. We compared recurrence rates before and after different points after complete resection. The LRR, DR, and overall recurrence rates after the first 6 months and 12 months significantly decreased among the NCRT group (eTable 4 in Supplement 2). The 5-year cumulative incidence of LR, DR, and overall recurrence in the NCRT group were 15.3%, 24.3%, and 32.2%, respectively, whereas those in the surgery group were 27.9%, 40.1%, and 50.9%, respectively (eTable 5 in Supplement 2).

## Discussion

After long-term follow-up, the final results from the NEOCRTEC_50_10 randomized clinical trial were consistent with the initial outcomes.^14^ The findings of the present study demonstrated that treatment with NCRT followed by surgery significantly improved OS and DFS among patients with locally advanced ESCC compared with surgery alone. Locoregional and distant disease control were both significantly improved by treatment with NCRT plus surgery. The phase 3 Chemoradiotherapy for Oesophageal Cancer Followed by Surgery Study (CROSS) phase 3 trial demonstrated that treatment with NCRT followed by surgery significantly prolonged OS among patients with esophageal or esophagogastric junction cancer compared with surgery alone.^4^ The OS benefit was also observed in the subgroup of patients with ESCC. The current study recruited 451 patients with locally advanced ESCC. To our knowledge, this is the largest-scale clinical trial comparing NCRT plus surgery with surgery alone among patients with ESCC. The findings of the NEOCRTEC_50_10 clinical trial confirm the results of the CROSS and support the importance of this combined treatment modality for patients with locally advanced ESCC.

Several previous randomized clinical trials have evaluated NCRT plus surgery vs surgery alone for the treatment of esophageal cancer.^4,6,7,8,9,10,11,12,13^ However, the results have been inconsistent, especially among patients with ESCC. A phase 3 clinical trial performed by Burmeister et al^10^ recruited 256 patients with esophageal or esophagogastric junction cancer, including 95 patients (37.1%) with ESCC and 217 patients (84.5%) with clinical N0 status. The results indicated that NCRT did not significantly improve progression-free survival or OS among patients with esophageal cancer in comparison with surgery alone. Another phase 3 clinical trial, the Francophone de Cancérologie Digestive 9901 (FFCD9901) study,^13^ enrolled 195 patients with stage I/II esophageal cancer, 137 of whom (70.3%) had ESCC. The results showed that the OS was comparable between the group receiving preoperative chemoradiotherapy plus surgery and the group receiving surgery alone. Moreover, postoperative mortality was significantly higher in the NCRT group compared with the surgery only group (11.1% vs 3.4%, respectively; *P* = .05).

In contrast to these clinical trials, the current study enrolled patients with locally advanced ESCC, 390 of 451 of whom (86.5%) had clinical N1 disease. In the CROSS, 236 of 366 patients (64.5%) had clinical N1 disease. These patients have a higher tumor burden and a higher risk of metastasis and would likely benefit most from the combined therapy. Moreover, the safety of the multimodal therapy is an important factor because toxic effects from chemoradiotherapy could counteract the survival benefit. In the current study, treatment with NCRT plus surgery did not significantly increase peritreatment mortality compared with surgery alone (2.2% vs 0.4%; *P* = .21).^14^ With regard to death in the hospital, the CROSS found no significant difference between the group receiving NCRT plus surgery and the group receiving surgery alone (3.6% vs 4.3%, respectively; *P* = .70).^19^ Therefore, the longer-term outcomes of both clinical trials showed that treatment with NCRT plus surgery significantly improved OS compared with surgery alone.

In the present study, further improvement in OS was found in the surgery group, and the results were better than those previously reported.^4,6,7,8,9,10,11,12,13^ Several reasons might have contributed to the favorable outcome. First, the protocol strictly adhered to performing 2-field lymphadenectomy with total mediastinal lymph node dissection. Second, the included population was relatively young. Third, the postoperative mortality rate was only 0.9%. Other randomized clinical trials conducted in Asia using similar pathologic staging distribution also reported similar long-term outcomes. For example, the surgery only group from the Japan Clinical Oncology Group 9204 (JCOG9204) clinical trial^20^ included 82.8% of patients with pathologic N1 disease and 55% of patients with stage III to IV disease. The 5-year OS rate of this group was 52%. Another randomized clinical trial conducted by Li et al^21^ compared long-term survival between right and left thoracic approaches, and this study also reported good 5-year OS rates (>40%) among patients with positive lymph nodes in the right thoracic group. Thus, our results are consistent with those of several previous studies. Moreover, the results of the present study indicate that the significant difference in OS may be ascribed to the effectiveness of treatment with NCRT rather than worse outcomes in the surgery group.

In the current study, the significant survival benefits of this multimodal therapy were attributed to several factors. First, treatment with NCRT plus surgery significantly reduced LRRs and distant metastases. Second, by tumor downstaging, the receipt of preoperative chemoradiotherapy compared with surgery alone significantly improved the R0 resection rate (98.4% vs 91.2%; *P* = .002),^14^ which is an independent positive prognostic factor.^22,23^ Third, among patients who underwent successful R0 resection compared with surgery alone, by decreasing lymph node metastases, treatment with NCRT was able to reduce lymph node involvement among patients (32.4% vs 63.8%; *P* < .001),^24^ which is a substantial negative prognostic factor.^24^ Fourth, neoadjuvant therapy may have eliminated micrometastasis early,^25,26,27^ which may account for disease recurrence.

### Limitations

This study has limitations. First, this multimodal therapy could not be readily applied to older patients or patients with lower performance status because of the lack of patients in these categories in the cohort. Second, the clinical trial was conducted in China, where there is a high prevalence of ESCC. Whether the proposed preoperative regimen is applicable to esophagogastric junction adenocarcinoma, which is more prevalent in Western countries, requires additional study. Third, among the 38 patients (17.0%) who did not receive surgery after chemoradiotherapy, 4 patients died while waiting to receive surgery. Most of those patients declined receipt of surgery after tumor response to chemoradiotherapy and relief of dysphagia. In accordance with the intention-to-treat principle, all of these patients were included in the survival analysis.

To date, there are few data from direct comparison of treatment with preoperative chemoradiotherapy vs preoperative chemotherapy. The Medical Research Council Esophageal Cancer Trial^28,29^ showed that treatment with preoperative cisplatin and fluorouracil chemotherapies significantly increased survival among patients with esophageal cancer compared with surgery alone. However, the Radiation Therapy Oncology Group 8911 clinical trial^30,31^ revealed that OS was comparable between treatment with preoperative chemotherapy plus surgery and surgery alone. The ongoing Japan Clinical Oncology Group 1109 NEXT (A Randomized Controlled Phase III Comparing CF vs DCF vs CF-RT as Neoadjuvant Treatment for Locally Advanced Esophageal Cancer) clinical trial may provide more evidence on the selection of neoadjuvant therapy for the treatment of patients with ESCC.^32^

## Conclusions

Treatment with NCRT according to the NEOCRTEC_50_10 regimen was found to significantly prolong long-term overall and disease-free survival among patients with locally advanced ESCC. Neoadjuvant chemoradiotherapy followed by surgical resection may be considered a standard of care for patients with potentially resectable locally advanced ESCC.

## Notes

Supplement 1.
Trial Protocol

Supplement 2. **eMethods.** Technique of Bilateral Recurrent Nerve Lymph Node Dissection When a Patient Underwent Ivor Lewis Esophagectomy **eTable 1.** Participating Centers **eTable 2.** Multivariable Cox Analysis for Disease-free Survival Stratified by Subgroups **eTable 3.** Number of Patients Receiving R0 Resection With Locoregional or Distant Recurrence **eTable 4.** Comparison of Number of Patients With Locoregional or Distant Recurrence According to Different Time Points During Follow-up **eTable 5.** 5-Year Cumulative Incidence of Locoregional Recurrence, Distant Recurrence, and Overall Recurrence

Supplement 3.
Data Sharing Statement
