# Supplementary material for: Long-term Efficacy of Neoadjuvant Chemoradiotherapy Plus Surgery for the Treatment of Locally Advanced Esophageal Squamous Cell Carcinoma: The NEOCRTEC5010 Randomized Clinical Trial
Source: JAMA Surg. 2021 Jun 23;156(8):721–9. doi: 10.1001/jamasurg.2021.2373 (PMC8223138; doi:10.1001/jamasurg.2021.2373)
Supplement: Supplement 1. — Trial Protocol [file jamasurg-e212373-s001.pdf]

# PREOPERATIVE CHEMORADIOTHERAPY PLUS SURGERY VERSUS SURGERY ALONE IN THE TREATMENT OF LOCALLY ADVANCED ESOPHAGEAL SQUAMOUS CELL CARCINOMA: A MULTICENTER, RANDOMIZED, CONTROLLED STUDY (NEOCRTEC5010)

**Cooperative Centers: Sun Yat-sen University Cancer Center; Shantou University Medical College Affiliated Cancer Hospital; Zhejiang Taizhou Hospital; Shanghai Jiaotong University Affiliated Chest Hospital; Tianjin Medical University Affiliated Cancer Hospital; Fudan University Affiliated Cancer Hospital, Shanghai; Zhejiang Cancer Hospital; Sichuan Cancer Hospital**

Clinical research protocol

Sun Yat-sen University "5010 Plan" project

Version number / date: 4.0 / 2015.12.01


**Principal investigators**

Professor Fu Jianhua
Department of Thoracic Surgery
Sun Yat-sen University Cancer Center
651 Dongfeng Road East, Guangzhou City
Tel: (020) -87343638; Fax: (020) -87343628;
E-mail: fujh@sysucc.org.cn

Professor Liu Mengzhong
Department of Radiation Oncology
Sun Yat-sen University Cancer Center
651 Dongfeng Road East, Guangzhou City
E-mail: liumzh@sysucc.org.cn


**Participating Centers**

Cancer Hospital of Shantou University Medical College, Shantou
Study Coordinator: Professor Chen Yu Ping

Department of Thoracic Surgery
Cancer Hospital of Shantou University Medical College
7Raoping Road, Shantou City, Guangdong Province
E-mail: stchenyp@hotmail.com

Taizhou Hospital of Zhejiang Province, Taizhou

Study Coordinator: Professor Zhu Chengchu
Department of Thoracic Surgery
Taizhou Hospital of Zhejiang Province
150 Ximen Street, Linhai City, Zhejiang Province
E-mail: zhucc@enzemed.com

Shanghai Chest Hospital, Shanghai Jiaotong University, Shanghai

Study Coordinator Professor Fang Wentao
Department of Thoracic Surgery
Shanghai Chest Hospital, Shanghai Jiaotong University
241WestHuaihai Road, Shanghai city
E-mail: vwtfang@hotmail.com

Tianjin Medical University Cancer Hospital, Tianjin

Study Coordinator Professor Yu Zhentao
Department of Thoracic Surgery
Tianjin Medical University Cancer Hospital
1 West Huanhu Road, Hexi District, Tianjin
E-mail: YUZHTAO@HOTMAIL.COM

Fudan University Shanghai Cancer Center, Shanghai

Study Coordinator Professor Xiang Jiaqing
Department of Thoracic Surgery,
Fudan University Shanghai Cancer Center
270 Dong’an Road, Xuhui District, Shanghai City.
E-mail：j.q.xiang@hotmail.com

Zhejiang Cancer Hospital, Hangzhou

Study Coordinator Professor Mao Weimin
Department of Thoracic Surgery
Zhejiang Cancer Hospital
38 Guangji Road, Banshang Bridge, Hangzhou City.

E-mail：maowm1218@163.com

Sichuan Cancer Hospital, Chengdu

Study Coordinator Professor Han Yongtao
Department of Thoracic Surgery
Sichuan Cancer Hospital
55 Four Section, Renmin South Road, Chengdu City.
E-mail：hanyongt@aliyun.com

**Grant**

1. The Health Ministry of China [grant number: 179]
2. Sun Yat-sen University Clinical Research 5010 Program [grant number: 2007048]
3. National Science Foundation of China [grant number: 81272635]
4. Science and technology fund for projects of Guangdong Province [grant number: 2012A030400007]
5. Zhejiang Provincial Science and Technology Major Projects [grant number: 2011C13039-2]

## SCHEMA

**Esophageal Cancer**
**Histology: squamous cell carcinoma**
**Staging: T1-4N1M0/T4N0M0 by the AJCC 6th edition**

↓

**REGISTRATION**

↓

**STRATIFICATION**
**(By center)**

↓

**RANDOMIZATION**

| **ARM 1** | **ARM 2** |
|---|---|
| Neo-adjuvant Chemoradiotherapy<br>+ Surgery | Surgery |
| Total Dose: 40.0 Gy<br>Dose / Fraction: 2.0Gy<br>Fraction / week: 5<br>Concomitant Vinorelbine 25mg/m$^2$ IV<br>Days 1, 8, 22 & 29<br>+ Cisplatin 75 mg/m$^2$ IV DRIP<br>Days 1 & 22<br>Or +Cisplatin 25mg/m$^2$ IV DRIP<br>Days 1, 2, 3, 4, 22, 23, 24 & 25 | McKeown esophagectomy<br>or Ivor Lewis esophagectomy |
| Surgery: same as ARM 2 | |

**Follow-up at least every 3 months in the first year and every 6 months thereafter**

## 1.0 BACKGROUND

Esophageal cancer (EC) is the eighth most common cancers in the world, with more than 480,000 new cases and 400,000 deaths occurred annually worldwide[1]. In China, every year, no matter new cases or deaths account for more than half of the world. Besides, over 90% of Chinese EC patients have esophageal squamous cell carcinoma (ESCC).

Surgery is the main treatment of this disease, but the prognosis of patients with locally advanced esophageal cancer is rather poor. As a result of surgery alone, the 5-year survival rate of about 25% has not changed significantly in several decades[2].

Preoperative chemoradiotherapy followed by surgery seems to hopefully improve the survival of EC. Nevertheless, the results of different studies were inconsistent[2]. The CROSS trial has demonstrated that preoperative chemoradiotherapy can significantly increase the overall survival of patients with EC compared with surgery alone[3]. It should be noticed that only 84 cases (23%) of ESCC were enrolled in this trial, which may be not perfect to evaluate the effect of this combined therapy for this tumor type. The FFCD9901 trial, which included 137cases (70.3%) of ESCC, has showed that there was no significant difference between the preoperative chemoradiotherapy and surgery alone group with respect to overall survival[4].

Up till now, vinorelbine has no indications for esophageal cancer, although, some studied have reported its effect and feasibility to the therapy of EC[5-7]. Vinorelbine has similar mechanism with paclitaxel and docetaxel, which are recommended for the chemotherapy of EC by NCCN. They are all classified as antimicrotubule agents, which cause mitotic arrest and eventual cell death through inhibition of microtubule dynamics. In comparison with the taxanes, vinorelbine has obvious advantage of little cardiac toxicity. This should be beneficial to prevent cardiac side effects of chemoradiotherapy, especially for the middle or lower thoracic EC, which account for over 70% of thoracic EC in China. For this group of patients, radiotherapy can hardly avoid cardiac toxicity.

Based on our preliminary study[8], we have demonstrated the validity and safety of vinorelbine and cisplatin-based neoadjuvant chemoradiotherapy.

We are to carry out a phased III clinical trial to investigate the effect of this multidisciplinary therapy for the overall survival of patients with locally advanced ESCC.

## 2.0 OBJECTIVE

The purpose of this study is to compare neo-adjuvant chemoradiotherapy followed by surgery

versus surgery alone, in terms of the overall survival time (OS) in patients with Stage IIB or III squamous cell esophageal carcinoma.

## 3.0 STUDYDESIGN

The NEOCRTEC5010 trial is a multicenter, randomized phase III, clinical trial. The study started on 1 July 2007 and the duration of inclusion will be approximately 7 years. The study compares neoadjuvant chemoradiotherapy followed by surgery with surgery alone in patients with potentially curable locally advanced esophageal squamous cell carcinoma, with inclusion of 215 patients per arm.

## 4.0 ENDPOINTS

The primary endpoint is overall survival. The time from the date of randomization to the date of death or the last follow-up will be calculated as overall survival (OS).
As to secondary endpoints, we aim to compare disease-free survival (DFS), safety, rate of R0 resection, and pathologic response. The time from the date of R0 resection to the date of disease recurrence or death will be calculated as disease-free survival (DFS).
The toxicity of chemotherapy and radiotherapy will be evaluated according to the National Cancer Institute Common Terminology Criteria for Adverse Events Version 3.0 (CTCAE v 3.0).Perioperatively, the following will be evaluated: type of procedure, surgical resection rate, operation time, blood loss, thoracic drainage fluid volume, hospital stay, incidence of complications, and perioperative mortality.

## 5.0 STAGING CRITERIA – THE 6th AJCC EDITION

Primary Tumor (T)

| | |
|---|---|
| TX | Primary tumor cannot be assessed. |
| T0 | No evidence of primary tumo |
| Tis | Carcinoma *in situ* |
| T1 | Tumor invades lamina propria or submucosa |
| T2 | Tumor invades muscularis propia |
| T3 | Tumor invades adventitia |
| T4 | Tumor invades adjacent structures |

Regional Lymph Nodes (N)

| | |
|---|---|
| NX | Regional lymph nodes cannot be assessed |
| N0 | No regional lymph node metastasis |
| N1 | Regional lymph node metastasis |

Distant Metastasis (M)

| | |
|---|---|
| MX | Distant metastasis cannot be assessed |
| M0 | No distant metastasis |
| M1 | Distant metastasis |

Tumors of the lower thoracic esophagus:

| | |
|---|---|
| M1a | Metastasis in celiac lymph nodes |
| M1b | Other distant metastasis |

Tumors of the mid-thoracic esophagus:

| | |
|---|---|
| M1a | Not applicable |
| M1b | Non-reginal lymph nodes and/or other distant metastasis |

Tumors of the upper thoracic esophagus:

| | |
|---|---|
| M1a | Metastasis in cervical nodes |
| M1b | Other distant metastasis |

Stage grouping

| | | | |
|---|---|---|---|
| Stage 0 | Tis | N0 | M0 |
| Stage I | T1 | N0 | M0 |
| Stage IIA | T2 | N0 | M0 |
| | T3 | N0 | M0 |
| Stage IIB | T1 | N1 | M0 |
| | T2 | N1 | M0 |
| Stage III | T3 | N1 | M0 |
| | T4 | Any N | M0 |
| Stage IV | Any T | Any N | M1 |
| Stage IV A | Any T | Any N | M1a |
| Stage IV B | Any T | Any N | M1b |

## 6.0 PATIENT SELECTION

### 6.1 Inclusion Criteria

a. Patients diagnosed with potentially resectable stage IIb or III thoracic esophageal squamous cell carcinoma (according to the 6th AJCC edition).
b. With no previous treatment.
c. With at least 6-month expected survival.
d. Patients aged from 18 to 70.
e. Adequate marrow: white blood cell≥$4.0\times10^9$/L; neutrophil ≥$1.5\times10^9$/L; platelet ≥$100.0\times10^9$/L; hemoglobin ≥90 g/L.
f. With normal liver and kidney function.
g. Satisfactory performance status: Karnofsky performance score (KPS) ≥ 90.
h. Informed consent will be obtained before the study.

### 6.2 Exclusion Criteria

a. Prior treatment to primary tumor or nodes.
b. With allergic history or suspicious allergy to chemotherapy agents such as DDP and Vinorelbine.
c. History of or concomitant hemorrhagic diseases.
d. Surgery is not allowed because of other uncontrollable diseases.
e. Pregnancy or lactation.
f. Patients are incapable of signing informed consent due to psychological, family or social reasons.
g. Reconstruction with stomach as the conduit are infeasible due to prior surgery.
h. Patients have peripheral neuropathy and the CTCAE v3.0 grade is ≥2.
i. Prior malignancies except for adequately treated basal cell or squamous cell skin cancer, in situ cervical cancer.

### 6.3 Criteria for Removal from Protocol Treatment

a. Distant metastasis is present during treatment.
b. Intercurrent disease which would affect assessments of clinical status to a significant degree, require discontinuation of drug, or both.
c. Unacceptable toxicity.
d. Patients become intolerant of surgery after preoperative CRT.
e. The patient may withdraw from the study at any time for any reason.

## 7.0 REGISTRATION GUIDELINES

7.1 All the patients must be registered with the coordinator of respective center prior to initiation of treatment.

7.2 Registration form and consent form will be submitted.

7.3 The randomization desk will confirm all eligibility criteria and obtain essential information (including stratification factors and patient number).

## 8.0 STRATIFICATION / RANDOMIZATION SCHEME

### 8.1 Stratification

Patients will be stratified according to the treatment centers *(SYSUCC vs. SUMCCH vs. TZH vs. SHCH vs. TJMUCH vs. FUSCC vs. ZJCH vs. SCH)*.

### 8.2 Randomization

Eligible patients will be randomized using a 1:1 allocation of patients to ARM1 and ARM2. Stratification factors for the randomization are centers defined in Section 8.1. The block sizes will be chosen by the statistician so that each block contains the patients in equal proportion. This procedure helps to ensure both randomness and investigator blinding (the block sizes are known only to the statistician), as recommended by Friedman et al[9].Randomization will be generated by the statistician in sealed envelopes labeled by stratum. Only after the registration, the envelopes will be opened. Every patient will have a corresponding unique subject number during the whole study.

Eligible patients will be randomized to either:

ARM 1: Preoperative Chemoradiotherapy followed by Surgery or

ARM 2: Surgery alone.

## 9.0 EXAMINATIONS AND SCREENING OF PATIENTS

### 9.1 Examinations

All the patients should receive the following examinations before recruitment within 7 days before treatments.

9.1.1 Complete medical history and systemic physical examinations (including symptoms, signs, body weight loss and KPS).

9.1.2 Routine examinations:

a. Routine blood tests (blood type, clotting time, hemoglobin, granulocyte count, platelet count, and granulocyte sorting).
b. Serological examinations: liver and kidney function, blood lipids, blood glucose, blood electrolytes, and tumor markers.
c. Electrocardiography.
d. Lung function tests.
e. Histopathology: pathological examination will be done based on the tissues from esophageal biopsy.
f. X-ray: anterior-posterior and lateral chest X-ray, esophageal barium.
g. Neck, chest and abdominal CT (plain and enhanced) from the first cervical spine to the umbilical plane.
h. Esophagogastroduodenoscopy (EGD), with ultrasound endoscopy (EUS).
i. Cervical ultrasonography.
j. If necessary, electronic bronchoscopy will be employed to confirm the involvement of trachea and/or bronchus.
k. If necessary, PET-CT will be employed to exclude distant metastasis.
l. If necessary, ECT will be employed to exclude bone metastasis.

### 9.2 Screening of patients

After examinations, patients are assessed according to the above-mentioned criteria, and those meeting the inclusion criteria and signing informed consent will be recruited into the study and will be randomized into the two treatment groups.

## 10.0 THERAPEUTIC PROTOCOLS

### 10.1 Protocol for patients receiving preoperative concurrent CRT and surgery (Figure 1)

10.1.1 Preoperative Chemotherapy

Patients will receive two cycles of vinorelbine and cisplatin. Vinorelbine, 25 $mg/m^2$,iv bolus, days 1 and 8. DDP, 75 $mg/m^2$, ivdrip, within 3hr,day 1 (or DDP, 25 $mg/m^2$, will be intravenously injected within 2hr, on days 1-4) every three weeks.

10.1.2 Pretreatment before chemotherapy

1) Antiemetic prophylaxis will be given. For acute vomiting on the first day, a 5-HT3 antagonist will be administered 1 hr before chemotherapy, and dexamethasone (DXM, 20 mg) will be administered orally or intravenously within 5-15 minutes afterward. For delayed vomiting that occurred on days 2-4, DXM, 8 mg BID and metoclopramide, 20 mg BID, will be administered for 3 consecutive days. Metoclopramide can be replaced with a 5-HT3 antagonist.
2) To increase hydration before administering cisplatin, patients will be asked to drink 2 L of water 1 day before chemotherapy. On day 1 with cisplatin 75 $mg/m^2$, 1000 mL of normal saline (NS) or glucose solution (GS) will be administered intravenously before chemotherapy, followed by cisplatin administered intravenously in 500 mL of normal saline. 5% oblimersen sodium (Genasense®; GNS) or 5% GS or NS will be administered intravenously at a rate of 200-250 mL/hr for 4-6 hr. While receiving cisplatin, patients will be hydrated with up to 3,000 mL of normal saline. If DDP is administered within 4 days, hydration will not be needed.
3) Patients will receive 20% mannitol (250 mL) as intravenous infusion before cisplatin, or 125 mL of 20% mannitol intravenous infusion before and after cisplatin. After DDP treatment, furosemide (40 mg) will be administered intravenously according to the patient's urine volume.

10.1.3 Preoperative radiotherapy

1) During CT simulation, patients will be immobilized with a vacuum bag in the supine position with arms raised. Radiation will be delivered by three-dimensional conformal radiotherapy (3DCRT) using 6-8 MV X-ray. The prescription dose to PTV should be 40 Gy in 20 fractions over 4 weeks, starting the first day of the first cycle of chemotherapy (*Figure 1*).
2) Gross tumor volume (GTV) is defined as the primary tumor and involved lymph nodes on CT and EUS. Clinical target volume (CTV) is defined as the GTV plus 3-cm proximal and distal margins and a radial margin of 0.5–1.0 cm, as well as elective nodal regions. Planning target volume is determined by adding 0.8 cm radially to the CTV.
3) The prescription dose will be specified at the International Commission on

Radiation Units and Measurements (ICRU) 50/62 reference point, which will usually be the isocenter. The isodose curve representing 93% of the prescription dose should encompass the entire PTV. Tissue density inhomogeneity correction will be performed. Radiation therapy will be delivered by the combination of anterior/posterior, oblique or lateral field. Customized blocks or a multi-leaf collimator will be used to shape the treatment fields. All patients will undergo a 3D planning. Beams-eye-view (BEV) displays will be used to ensure optimal target volume coverage and optimal normal tissue sparing.

4) Organs at risk (OARs) include both lungs, heart, spinal cord, and liver. Dose-Volume-Histograms (DVHs) will be used to select the most appropriate treatment plan and to evaluate the normal tissue damage. Standard dose constraints are applied for treatment plan: mean lung dose <17 Gy, total lung volume receiving greater than 20 Gy (V20) of <30%, heart V40 <30%, liver V30 <30%, and maximum spinal cord dose <45 Gy. Every effort should be made to keep the total lung dose to a minimum.

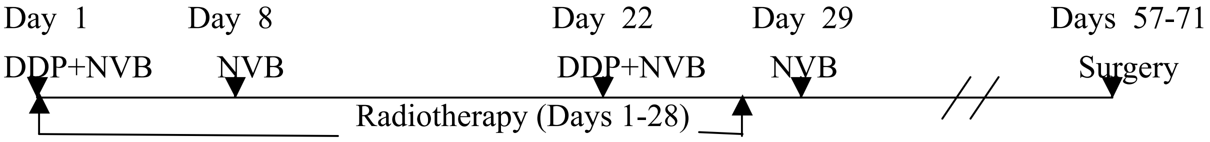


*Figure 1 Time schedule of preoperative CRT*

10.1.4 Criteria for radiation toxicity

1) The National Cancer Institute Common Terminology Criteria for Adverse Events v3.0 (CTCAE v3.0) is used for assessment of toxicity during radiotherapy.
2) Acute and late toxicity related to radiation therapy include fatigue, myelosuppression, skin erythema, subcutaneous fibrosis, esophagitis, carditis, myelitis, and radiation pneumonitis.
3) In some cases, medical support and/or a feeding tube will be necessary. When grade 4 radiation-related toxicity is observed, active symptomatic treatment will be administered and radiotherapy will be withheld until the toxicity has recovered to Grade 3.
4) If any of the following toxicity is present, patients will be excluded from the treatment protocol: esophageal perforation and heavy hemorrhage, non-healing esophageal tracheal leakage, myocardial infarction, heart failure, severe arrhythmias, and radiation pneumonia with dyspnea.
5) If interruption of therapy (up to two weeks) becomes necessary, radiotherapy should be completed to the prescribed doses. Total number of fractions and

elapsed days should be carefully reported. If an interruption of more than two weeks is necessary, resumption of treatment is at the discretion of the radiation oncology chairs. The patient will be considered a major deviation, but follow-up will be continued.

10.1.5 Principles for the adjustment of dosage of chemotherapeutic agents

1) The CTCAE v3.0 guidelines will be used to evaluate toxicity from chemotherapy.
2) The dosage of chemotherapeutic agents will be adjusted according to the toxicity of the first course of chemotherapy (*Table 1*).

**Table 1. Adjustment of chemotherapy dosage during the second course of chemotherapy**

| **The most severe toxicity in the first course of chemotherapy (CTCAE v 3.0)** | **Vinorelbine** | **Cisplatin** |
|---|---|---|
| **Hematologic toxicity** | | |
| Granulocyte deficiency-related fever (body temperature ≥38.5 and grade 3/4 neutropenia regardless of duration) | 75% of standard dose | 75% of standard dose |
| Grade 4 thrombocytopenia or grade 3 thrombocytopenia with hemorrhage | 75% of standard dose | 75% of standard dose |
| **Gastrointestinal reactions** | | |
| Grade 3 nausea/vomiting after prophylactic antiemetic treatment or symptomatic treatment | 75% of standard dose or unchanged | 75% of standard dose or unchanged |

| | | |
|---|---|---|
| Grade 4 nausea/vomiting after prophylactic antiemetic treatment or symptomatic treatment | 50% of standard dose | 50% of standard dose |
| **Other non-hematological toxicity** | | |
| Any grade 3 toxicity | 75% of standard dose | 75% of standard dose |
| Any grade 4 toxicity | 50% of standard dose or discontinuation of treatment | 50% of standard dose or discontinuation of treatment |
| **Neurological toxicity** | | |
| Grade 2 | 50% of standard dose (no delayed administration) | 50% of standard dose (no delayed administration) |
| Grade 3 or 4 | Treatment suspended for up to 2 weeks; 50% of standard dose if improvement to grade 2 toxicity, or treatment discontinued | Treatment suspended for up to 2 weeks; 50% of standard dose if improvement to grade 2 toxicity, or treatment discontinued |
| **Renal toxicity** | | |
| Creatinine clearance≥60 ml/min | Unchanged | Unchanged |
| 50≤Creatinine clearance <60 ml/min | Unchanged | 75% of standard dose |
| Creatinine clearance <50 ml/min | Unchanged | Discontinue cisplatin. |

10.1.6 Criteria for next course of chemotherapy:

a. ANC≥1.5×$10^9$/L
b. Platelet count ≥100×$10^9$/L
c. ALT, AST and total bilirubin ≤CTCAE v3.0 grade 2
d. Non-hematologic toxicity (except for hair loss) returned to grade 1 or baseline level
e. Neurological toxicity ≤CTCAE v3.0 grade 2

10.1.7 Delayed chemotherapy

Chemotherapy will be delayed if, after a course of chemotherapy, the patient has failed to meet the criteria for the next course, and treatment will then be suspended for up to 2 weeks. Then, after 2 weeks, treatment will be halted if patients still do not meet the criteria for the next course of chemotherapy. Before the next course of chemotherapy, patients will undergo relevant laboratory examinations once a week and their recovery will be evaluated by the investigators. Chemotherapy will be reinstated if patients recover and meet the criteria for the next course of chemotherapy. The chemotherapeutic dosage will be adjusted if necessary.

10.1.8 Regular evaluation during and after preoperative CRT

Regular evaluation will consist of: routine blood tests once a week; liver and kidney function before and after chemotherapy; esophageal barium x-ray examination after the patient received radiotherapy with 20 Gy and 40 Gy. All patients underwent clinical re-staging approximately 4-6 weeks after the completion of CRT, including physical examination, standard laboratory tests, EGD with EUS, pulmonary function tests, Esophageal barium x-ray, and neck/chest/abdominal CT with contrast.

10.1.9 Timing of surgery

Approximately 4–6 weeks after completion of CRT, surgery will be performed if the patient's white blood cells, platelets, and liver and kidney function have all returned to normal. Routine blood tests, blood biochemical examinations, chest and abdominal CT scans, detection of lung function, and electrocardiograms will be performed before surgery.

10.1.10 Surgical procedures

The two groups undergo the same surgical procedure specified as followed.

## 10.2 Protocol for patients receiving surgery alone

Surgery will be conducted  soon after the randomization. McKeown or Ivor Lewis esophagectomy will be performed. Two-field lymphadenectomy with total mediastinal lymph node dissection is performed during sugery. Radical resection will be defined by both macroscopic observation and postoperative pathological negative margin. A jejunum tube will be placed via the nose or by jejunostomy.

The lymph nodes resected during total lymphadenectomy include the left recurrent laryngeal nerve nodes, right recurrent laryngeal nerve nodes, infraaortic arch nodes, periesophageal nodes of the upper, middle and lower thoracic portion, the infracarinal nodes, the posterior mediastinal nodes, the paracardiac nodes, lesser curvature nodes, the left gastric nodes, the common hepatic nodes, the splenic nodes and the celiac nodes.

### 10.3 Pathology

The resection specimens will be macroscopically and microscopically reviewed by a team of experienced pathologists using the standard protocol (margins, tumor type and extension, lymph nodes). The lymph node dissection should contain at least 15 nodes. For patients receiving preoperative CRT, the pathological response will be defined according to the tumor regression grade, as described by Mandard et al. In the absence of macroscopic tumor, the whole resected tissue was embedded in paraffin to make an assessment for the presence of any residual tumor. Pathologic complete response (pCR) was defined as the absence of residual tumor within the esophagus and lymph nodes.

The pathology report should contain the following: site of the tumor/lesion, type and grade of the tumor, extension into the esophageal wall, resection margins, therapy effects (Mandard score), lymph node status including the site, and the number of nodes with therapy effects.

Tumor Response Grading Systems (Mandard score)

Grade 1: Complete regression;

Grade 2: Isolated cell nests;

Grade 3: More residual cancer cells but fibrosis still predominates;

Grade 4: Residual cancer outgrowing fibrosis;

Grade 5: Absence of regressive changes.

## 11.0 <u>ETHICS</u>

### 11.1 Informed consent

Before patients recruitment, investigator should completely and comprehensively explain the objective of this study, the characteristics of drugs, and the potential toxicity and risk in the treatment, and allow the patients to be aware of their rights, risks and benefits. Informed consent form should be signed before recruitment and preserved in files as paper documentation.

### 11.2 Ethics and policy

This study will be conducted according to the Declaration of Helsinki (2000), Good Clinical Practice (GCP) published by CFDA and other relevant regulations. The study must be approved by the Ethics Committee from leading center and each participating institution. Any amendments of the study protocol should be re-approved by the Ethics Committee during the study.

## 12.0 DATA PROCESSING AND PRESERVATION

### 12.1 CRF

CRF (Case Report Form) should be timely filled in to assure accuracy and timely summary. CRF should not be obliterated. If there is mistake, the investigator should sign at the site of obliteration (see the introductions to filling in CRF). Three copies of CRF should be obtained to turn over to the leading site, sponsor and local site after the study. Data will be input into database after reviewing by the CRA. Then all of data cannot be altered.

### 12.2 Establishment of database

Once the CRF is received by the statisticians, the queries about the data should be answered by communicating with the investigator who fills in the CRF. The statisticians should timely establish database which is then reviewed and locked by the major investigators, sponsor, statisticians and CRA. Irrelevant persons are not accessible to the database and unable to alter the database. All the data should be backed up.

### 12.3 Preservation of materials

The investigators should preserve the documentations properly. According to the GCP, the documentations should be preserved by the investigators for more than 5 years.

## 13.0 STATISTICAL ANALYSIS

All patients will be included in the analysis whether they complete the treatment protocol or not. Data analyses will be done according to the 'Intention to treat' principle.

### 13.1 Sample size

The primary endpoint in this study is overall survival. We have the following set of hypotheses:

$H_0$: The study group has the same median survival of 39 months as the Control group;

$H_1$: The median survival of the study group and control group are different.

Sample size are calculated according to a projected median survival of 56 months and 39 months for patients assigned to multimodality treatment arm and surgery alone arm, respectively. We assumed an overall two-sided significance level of 5% and a statistical power of 80%. The randomization ratio between the experimental and control arms is 1:1. With 7 years' accrual, 2 years' follow-up, and two planned interim analyses, the calculated sample size will be 392 patients, 196 patients per arm[10]. Assuming that 10% will drop-out, the intended number of randomized patients will be 430, 215 per arm. Two interim analyses by an independent safety and data monitoring committee will be done after 107 patients (25%), and 430 patients (100%) have been enrolled.

### 13.2 Analysis of the primary endpoint

Overall survival (OS) is defined as the time period from the date of randomization to the date of death or the last follow-up. OS including 3-year and 5-year survival rates will be calculated using the Kaplan-Meier method, and then compared by the log-rank test.

### 13.3 Analyses of secondary endpoints

Disease-free survival (DFS) is defined as the time period from the date of R0 resection to the date of disease recurrence or death will be calculated as disease-free survival (DFS). DFS will be calculated using the Kaplan-Meier method, and then compared by the log-rank test. The rate of R0 resection, incidence of complications, and peritreatment mortality will be compared with the Chi-square test (or Fisher's exact test, if indicated).

### 13.4 *P* value

According to the O'Brien-Fleming algorithm, the two-sided significance level will be 0.000527 in the first interim analysis and 0.014 in the second interim analysis. The final significance level will be 0.045.

### 13.5 Interim analysis

13.5.1 An independent Data Monitoring Committee will be appointed to monitor the study and make decisions regarding possible early closure and publication.

13.5.2 The Committee will meet at least once a year (or discuss via electronic means) to ensure no undue toxicity and to monitor the data quality and results. Only this committee will be allowed access to the data until the decision is made to release the results.

13.5.3 Two formal interim analyses will be performed on 1 June, 2011 and 31 December, 2015.

13.5.4 The interim analysis of survival and toxicities will be reviewed by the committee. These results will not be the sole criteria for deciding whether to terminate accrual or report results early. All decision will also take into account the characteristics of patients, the nature of any toxicity, relevant external results, and other pertinent information.

13.5.5 Reports of the results will be made corresponding to the required number of events in the sample size calculation section of the protocol (about universal 2-year follow-up), and again after universal 5-year follow-up.

13.5.6 An Executive Committee composing of all the study coordinators will meet (or discuss via electronic means) every 6 months to monitor the patient accrual rate and to solve practical problems encountered during conduction of the trial.

13.5.7 Earlier and more frequent meetings will be called if excessive toxicities are observed.

## 14.0 FOLLOW-UP

The full-time study nurses will be responsible for the registration, follow-up of patients and inputting data of CRF. Then they will submit the information to the central office for further analysis. Each patient should have their own archive for evaluation (CRF), and a full-time study nurse will be responsible for the follow- up of each patient, as well as registration, inputting and preservation of information.

After the treatment has ended, patients will be re-examined in the hospital clinics once every 3 months within 1 year. Thereafter they will have regular follow-up visits once every 6 months until their deaths or the study ends. Physical examination, tumor markers, chest x-ray, Esophageal barium x-ray, cervical/abdominal ultrasonography will be performed at follow-up visits. Neck/chest/abdominal CT with contrast and EGD will be performed once every year.

**Note:** Patients who withdraw from this treatment protocol will also be followed up, and included for statistical analysis. History of the study protocol changes are shown in the *Appendix*.

## 15.0 BIBLIOGRAPHY


1. Jemal A, Bray F, Center M M, et al. Global cancer statistics.[J]. Ca A Cancer Journal for Clinicians, 2011, 61(2):69.
2. Herskovic A, Russell W, Liptay M, et al. Esophageal carcinoma advances in treatment results for locally advanced disease: review[J]. Annals of Oncology Official Journal of the European Society for Medical Oncology, 2012, 23(5):1095.
3. van Hagen P,Hulshof M C C M,van Lanschot J J B et al. Preoperative chemoradiotherapy for esophageal or junctional cancer.[J] .N. Engl. J. Med., 2012, 366(22): 2074-84.
4. Mariette C, Dahan L, Mornex F, et al. Surgery Alone Versus Chemoradiotherapy Followed by Surgery for Stage I and II Esophageal Cancer: Final Analysis of Randomized Controlled Phase III Trial FFCD 9901[J]. Journal of Clinical Oncology Official Journal of the American Society of Clinical Oncology, 2014, 32(23):2416.
5. Kulke M H, Muzikansky A, Clark J, et al. A Phase II Trial of Vinorelbine in Patients with Advanced Gastroesophageal Adenocarcinoma[J]. Cancer Investigation, 2006, 24(4):346.
6. Airoldi M, Cortesina G, Giordano C, et al. Docetaxel and vinorelbine: an effective regimen in recurrent squamous cell esophageal carcinoma.[J]. Medical Oncology, 2003, 20(1):19-24.
7. Koussis H, Scola A, Bergamo F, et al. Neoadjuvant carboplatin and vinorelbine followed by chemoradiotherapy in locally advanced head and neck or oesophageal squamous cell carcinoma: a phase II study in elderly patients or patients with poor performance status[J]. Anticancer Research, 2008, 28(2B):1383-1388.

8. Yang H, Fu J H, Hu Y, et al. [Neo-adjuvant chemoradiotherapy followed by surgery in treatment of advanced esophageal carcinoma][J]. Zhonghua Yi Xue Za Zhi, 2008, 88(45):3182.
9. Friedman L M, Furberg C D, Demets D L. Fundamentals of Clinical Trials[J]. Biometrics, 2010, 82(397):1055-1056.
10. Chow S C, Shao J, Wang H. Sample size calculations in clinical research[M]// Sample size calculations in clinical research/. Taylor & Francis, 2003:469-469.

**Duration of study and annual plan**

| | |
|---|---|
| 06, 2007 to 12, 2014 | Patients' recruitment and treatment |
| 01, 2015 to 12, 2019 | Follow up and data collection |

Appendix

**Modifications of the Study Protocol**

| Date | Before modification | After modification | Location |
|---|---|---|---|
| October 20, 2011 | 6.1 Inclusion criteria<br>d. Patients aged from 18 to 60 | 6.1 Inclusion criteria<br>d. Patients aged from 18 to 70 | P8 |
| October 20, 2011 | 13.5 Interim analysis<br>13.5.1 The Office of Good Clinical Practice from Sun Yat-sen University Cancer Center will monitor the study and make decisions regarding possible early closure and publication Interim analysis. | 13.5 Interim analysis<br>13.5.1 An independent Data Monitoring Committee will be appointed to monitor the study and make decisions regarding possible early closure and publication. | P19 |
| June 26, 2013 | 13.1 Sample size: 640 patients should be enrolled | 13.1 Sample size: 430 patients should be enrolled | P18 |
| December 1, 2015 | 13.3 Analyses of secondary endpoints<br>The rate of R0 resection will be compared by the t test. | 13.3 Analyses of secondary endpoints<br>The rate of R0 resection will be compared by the Chi-square test. | P18 |
