# Supplementary material for: Long-term Efficacy of Neoadjuvant Chemoradiotherapy Plus Surgery for the Treatment of Locally Advanced Esophageal Squamous Cell Carcinoma: The NEOCRTEC5010 Randomized Clinical Trial
Source: JAMA Surg. 2021 Jun 23;156(8):721–9. doi: 10.1001/jamasurg.2021.2373 (PMC8223138; doi:10.1001/jamasurg.2021.2373)
Supplement: Supplement 2. — eMethods. Technique of Bilateral Recurrent Nerve Lymph Node Dissection When a Patient Underwent Ivor Lewis Esophagectomy eTable 1. Participating Centers eTable 2. Multivariable Cox Analysis for Disease-free Survival Stratified by Subgroups eTable 3. Number of Patients Receiving R0 Resection With Locoregional or Distant Recurrence eTable 4. Comparison of Number of Patients With Locoregional or Distant Recurrence According to Different Time Points During Follow-up eTable 5. 5-Year Cumulative Incidence of Locoregional Recurrence, Distant Recurrence, and Overall Recurrence [file jamasurg-e212373-s002.pdf]

Supplementary Online Content

Yang H, Liu H, Chen Y, et al. Long-term efficacy of neoadjuvant chemoradiotherapy plus surgery for the treatment of locally advanced esophageal squamous cell carcinoma: the NEOCRTEC$_{50}$10 randomized clinical trial. *JAMA Surg*. Published online June 16, 2021. doi:10.1001/jamasurg.2021.2373

eMethods. Technique of Bilateral Recurrent Nerve Lymph Node Dissection When a Patient Underwent Ivor Lewis Esophagectomy

eTable 1. Participating Centers

eTable 2. Multivariable Cox Analysis for Disease-free Survival Stratified by Subgroups

eTable 3. Number of Patients Receiving R0 Resection With Locoregional or Distant Recurrence

eTable 4. Comparison of Number of Patients With Locoregional or Distant Recurrence According to Different Time Points During Follow-up

eTable 5. 5-Year Cumulative Incidence of Locoregional Recurrence, Distant Recurrence, and Overall Recurrence

This supplementary material has been provided by the authors to give readers additional information about their work.

© 2021 Yang H et al. *JAMA Surgery.*

eMethods. Technique of Bilateral Recurrent Nerve Lymph Node Dissection When a Patient Underwent Ivor Lewis Esophagectomy

During the thoracic part of Ivor Lewis esophagectomy, the surgeon firstly carried out the right recurrent laryngeal nerve (RLN) lymph node dissection. Mediastinal pleura was dissected along the right vagus nerve and posterior margin of the right subclavian artery. At the junction of the vagus nerve and the subclavian artery, the root of the right RLN could always be exposed. The surgeon bluntly freed the right RLN as long as possible. Ultrasound knife and scissors were used to do the skeletonization of RLN. With clear exposure of RLN, en bloc resection of the right RLN lymph nodes was completed.

The esophagus was then circumferentially mobilized from the esophageal hiatus to below the thoracic inlet. The azygos vein arch was isolated and divided. A retractor was used to press the trachea anteriorly, and the esophagus was pulled posteriorly. The surgeon separated and fully exposed the left margin of the trachea and the left main bronchus. The lymph nodes and soft tissues along the left RLN were separated from the very bottom of the posterior space of trachea. After separation of soft tissue at the level of the inferior segment of the trachea, the root of left RLN could always be observed at the level of the aorta arch. The surgeon bluntly freed the left RLN, and dissected the RLN lymph nodes along the left RLN.

© 2021 Yang H et al. *JAMA Surgery.*

eTable 1. Participating Centers

**Center**	**Principal Investigator**	**No. of enrolled patients**
Sun Yat-sen University Cancer Center	Prof· Jianhua Fu	203
Cancer Hospital of Shantou University Medical College	Prof· Yuping Chen	93
Taizhou Hospital, Wenzhou Medical University	Prof· Chengchu Zhu	66
Shanghai Chest Hospital, Shanghai Jiaotong University	Prof· Wentao Fang	58
Tianjin Medical University Cancer Hospital	Prof· Zhentao Yu	20
Zhejiang Cancer Hospital	Prof· Weimin Mao	4
Fudan University Shanghai Cancer Center	Prof· Jiaqing Xiang	4
Sichuan Cancer Hospital	Prof· Yongtao Han	3

© 2021 Yang H et al. *JAMA Surgery.*

eTable 2. Multivariable Cox Analysis for Disease-free Survival Stratified by Subgroups

	Group NCRT (events/n)	Group S (events/n)	HR (95% CI)	*P* value for interaction
All patients	77/182	123/207	0.57 (0.43, 0.76)	
Age				0.970
≤60	57/138	83/141	0.56 (0.40, 0.80)	
>60	20/44	40/66	0.58 (0.33, 1.01)	
Sex				0.076
Male	70/154	94/159	0.65 (0.48, 0.89)	
Female	7/28	29/48	0.29 (0.12, 0.69)	
Tumor location				0.861
Proximal third	10/20	13/19	0.39 (0.15, 1.01)	
Middle third	51/128	79/144	0.62 (0.44, 0.89)	
Distal third	16/34	31/44	0.48 (0.25, 0.93)	
Clinical T stage				0.605
T1-2	12/32	19/35	0.70 (0.33, 1.49)	
T3	44/105	79/134	0.54 (0.37, 0.79)	
T4	21/45	25/38	0.49 (0.26, 0.90)	
Clinical N stage				0.340
N0	9/22	15/24	0.37 (0.14, 1.02)	
N1	68/160	108/183	0.59 (0.44, 0.81)	

Abbreviations: NCRT, neoadjuvant chemoradiotherapy; S, surgery alone.

© 2021 Yang H et al. *JAMA Surgery.*

eTable 3. Number of Patients Receiving R0 Resection With Locoregional or Distant Recurrence

	**Group NCRT (n=182)**	**Group S (n=207)**	**HR (95% CI）**	***P* value**
Locoregional recurrence	25 (13.7)	45 (21.7)	0.53 (0.33, 0.87)	.01
Distant recurrence	46 (25.3)	74 (35.7)	0.60 (0.41, 0.86)	.006
Overall recurrence	63 (34.6)	102 (49.3)	0.60 (0.44, 0.82)	.001

Data are presented as n (%) unless specified. Locoregional, distant, and overall recurrences were referred to the interval from the date of surgery (R0 resection) to the date of first confirmation of recurrence. Deaths without recurrence were censored. The univariate cox model was used to analyze the recurrence pattern. Abbreviations: NCRT, neoadjuvant chemoradiotherapy; S, surgery alone.

© 2021 Yang H et al. *JAMA Surgery.*

eTable 4. Comparison of Number of Patients With Locoregional or Distant Recurrence According to Different Time Points During Follow-up

	Group NCRT (n=182)		Group S (n=207)		HR (95% CI)	*P* value
	events	%	events	%		
**Locoregional recurrence**						
During the first 6 months	5	2.7	10	4.8	1.28 (0.43, 3.81)	0.654
After first 6 months	20	11.0	35	16.9	**0.53 (0.31, 0.92)**	**0.023**
During the first 12 months	13	7.1	19	9.2	0.95 (0.46, 1.94)	0.882
After first 12 months	12	6.6	26	12.6	**0.41 (0.21, 0.80)**	**0.009**
During the first 24 months	15	8.2	33	15.9	0.86 (0.46, 1.59)	0.627
After first 24 months	10	5.5	12	5.8	0.69 (0.30, 1.59)	0.379
During the first 36 months	21	11.5	37	17.9	0.91 (0.53, 1.56)	0.736
After first 36 months	4	2.2	8	3.9	0.40 (0.12, 1.34)	0.138
**Distant recurrence**						
During the first 6 months	7	3.8	15	7.2	1.33 (0.52, 3.41)	0.555
After first 6 months	39	21.4	59	28.5	**0.62 (0.41, 0.92)**	**0.019**
During the first 12 months	22	12.1	34	16.4	0.82 (0.48, 1.42)	0.485
After first 12 months	24	13.2	40	19.3	**0.52 (0.32, 0.87)**	**0.012**
During the first 24 months	29	15.9	55	26.6	0.98 (0.62, 1.55)	0.933
After first 24 months	17	9.3	19	9.2	0.74 (0.39, 1.43)	0.371
During the first 36 months	36	19.8	65	31.4	0.88 (0.59, 1.33)	0.556
After first 36 months	10	5.5	9	4.3	0.91 (0.37, 2.24)	0.837
**Overall recurrence**						
During the first 6 months	12	6.6	22	10.6	1.51 (0.72, 3.13)	0.273
After first 6 months	51	28.0	89	43.0	**0.59 (0.42, 0.85)**	**0.004**
During the first 12 months	34	18.7	46	22.2	0.95 (0.61, 1.49)	0.831
After first 12 months	29	15.9	56	27.1	**0.45 (0.29, 0.71)**	**<0.001**
During the first 24 months	43	23.6	75	36.2	1.07 (0.73, 1.56)	0.746
After first 24 months	20	11.0	27	13.0	0.61 (0.34, 1.09)	0.097
During the first 36 months	52	28.6	88	42.5	0.95 (0.67, 1.34)	0.758

© 2021 Yang H et al. *JAMA Surgery.*

After first 36 months	11	6.0	14	6.8	0.64 (0.29, 1.41)	0.270

© 2021 Yang H et al. *JAMA Surgery.*

eTable 5. 5-Year Cumulative Incidence of Locoregional Recurrence, Distant Recurrence, and Overall Recurrence

	**Group NCRT (n=182)**	**Group S (n=207)**
5-year LR cumulative incidence, %	15.3(10.5-22.0)	27.9(21.4-35.9)
5-year DR cumulative incidence, %	24.3(18.4-31.6)	40.1(34.6-49.5)
5-year overall recurrence cumulative incidence, %	32.2(25.9-39.7)	50.9(44.0-58.2)

Abbreviations: NCRT, neoadjuvant chemoradiotherapy; S, surgery alone; LR, locoregional recurrence; DR, distant recurrence.

© 2021 Yang H et al. *JAMA Surgery.*
