# Supplementary material for: Long-term Efficacy of Neoadjuvant Chemoradiotherapy Plus Surgery for the Treatment of Locally Advanced Esophageal Squamous Cell Carcinoma: The NEOCRTEC5010 Randomized Clinical Trial
Source: JAMA Surg. 2021 Jun 23;156(8):721–9. doi: 10.1001/jamasurg.2021.2373 (PMC8223138; doi:10.1001/jamasurg.2021.2373)
Supplement: Supplement 3. — Data Sharing Statement [file jamasurg-e212373-s003.pdf]

Data Sharing Statement

Yang. Long-term Efficacy of Neoadjuvant Chemoradiotherapy Plus Surgery for the Treatment of Locally Advanced Esophageal Squamous Cell Carcinoma. *JAMA Surg*. Published June 16, 2021. doi:10.1001/jamasurg.2021.2373

Data
Data available: No
